# Rupture of the Membrana Tympani by Injection of Warm Water into the Auditory Canal for the Removal of Impacted Cerumen

**Published:** 1891-02

**Authors:** S. Latimer Phillips

**Affiliations:** Savannah, Ga.


VOL. VII. FEBRUARY, 1891. NO. 12.

### Original Communications.

### RUPTURE OF THE MEMBRANA TYMPANI BY INJECTION OF WARM WATER INTO THE AUDITORY CANAL FOR THE REMOVAL OF IMPACTED CERUMEN.

BY S. LATIMER PHILLIPS, M. D., OF SAVANNAH, GA.

In examining into the causes of injury to the membrana tympani, we find that it is very rarely produced by syringing the canal. It is because this accident is so unusual that I have seen fit to report the following case. Sudden forcing of the imprisoned air of the canal down against the frail membrana tympani, when the Eustachian tube is not sufficiently pervious to allow of an outlet to the air contained in the tympanum, will induce rupture of this membrane, on the same principle that we administer a blow on a bladder filled with air ; there being no room for expansion the membrane gives way at its weakest point, usually with a more or less loud sound. One of the most prolific causes of rupture of the drum-head is a sudden blow or slap upon the ear; the membrane is forced inwards against an immovable wall of air, and the result is rupture. Violent explosions, discharge of fire arms close to the ear, act upon the same principal. Direct pressure of a foreign body upon the membrane, as also fracture of the temporal bone, give a certain percentage of the causes.

Living as I do near to the seashore, I see every summer the usual contingent of patients with aural affections, the sequence to surf bathing. Now and then one will have a broken drum-head, the result of water striking the ear with considerable force, but the majority of perforations of the membrane follow an inflammation of the tympanum caused by the irritating briny water comingin contact with the delicate membrana tympani. Many of these people, ignorant of the danger of the surf to the ears, rush into the breakers without any protection to these delicate organs whatever, and carelessly expose them, broadside on, to the powerful force of the water. It is not surprising that once in a while one gets a broken drum to remember it by. At the time of the injury, a painful feeling in the ear may be experienced, with deafness later. On inspection, when rupture is the immediate sequence of the blow, a split in the membrane is found. In these cases of traumatic rupture of the drum-head, the tear is usually found in the near neighborhood of the umbo, and especially at the extremity of the bony process of the malleus running downward and backward, or downward and forward, following the course of the radiating fibers of the membrane, and frequently extending up to the bony insertion of the latter. When we have the opportunity of examining these cases shortly after the injury has been received, we find the split with probably a drop of blood in the canal or scattered over the drum surface in the near neighborhood of the opening. The hole will possibly be gaping from the drawing of the circular fibers and the healthy membrane of the tympanum showing through. If we do not see the case at the beginning, when we do, it will present a different picture. The opening will be larger and its edges irregular, with a discharge of a mucous or muco-purulent nature, the membrane behind swelled and thickened.

The case I have to report is that of a man of 54 years, in fairly good health, save when suffering from bleeding piles. For some weeks he had experienced dullness of hearing on left side, and wished, if possible, to be relieved. On inspection, canal of that side was found to be of average size, and containing a considerable amount of hardened wax. Hearing here was tick of watch at sixteen inches. Right ear normal as to hearing. With a small rubber ball syringe, holding about an ounce and a half of water, which I find to answer every purpose in washing the ear, and not capable of sending such a powerful stream as the larger metallic syringes, filled with luke-warm water, I began to syringe the auditory canal. Several syringefuls having been forced gently into the ear and not bringing the wax, I used a little more pressure. Suddenly something seemed to give way. Water poured from the patient's left nostril, and he complained of a peculiar sensation in that ear, though he had no pain worth speaking of. The wax was loosened and easily removed from the canal. I thought, of course, I would find a perforation of considerable size in the membrane, the sequel of an old purulent inflammation of the tympanum, as sometimes wax forms over the opening, mixing with the secretions from the diseased mucous membrane making quite a hard impervious wall to the secretions behind. When I looked at the drum-head I did not find what I expected, but one almost intact. It was congested in every part save the posterior superior quadrant. From the top of the malleus running down and back to the bony wall was a thin line of blood indicating the course of the rupture, and scattered here and there in its immediate region, a few drops of blood, which were easily wiped off with a piece of cotton wool. At this time there was no gaping, and even with Seigle's otoscope I could not make the wound gape. The hearing was found normal when tested with the watch and the Eustachian tube freely open. Previous to this attack he had never had deafness, nor had he ever any discharge from his ear. An antiseptic cotton wool plug was placed in the canal and directions to use warm water in the ear if he had any pain. When he returned on the morrow he had had no pain, but hearing in the affected ear had been reduced to watch tick at fifteen inches, less than he had before removal of wax. There was some mucous discharge and the opening in the membrane was wedged-shaped with its apex at tip of malleus, through which could be seen the swelled and congested mucous membrane lining the drum. A few drops of 95 per cent. alcohol were put in ear followed by slight burning. At night he noticed tinnitus in that ear. Without any other treatment hearing began to slowly improve. About this time he had an attack of malarial fever and was treated with quinine though I had advised him not to use it. In three weeks' time had taken 240 grains. After recovery from fever had an operation for removal of hemorrhoids. Two months from the time he first consulted me I saw him again. The opening had closed leaving only a thin line of scar, and hearing had become normal, he experiencing no inconvenience. Several months afterward the membrane gave way at this point from too vigorous blowing of the nose, but the parts soon united without any treatment whatever than a piece of cotton wool worn in the canal for a while, since which time he has been all right. In looking over the literature of the subject at my command, I find but one case where rupture of the drum membrane took place from syringing the canal. It is reported by Sexton (The Ear and Its Diseases, p. 221). A male child ten years old had his ear washed out by a physician for eczema with a large syringe used to administer enemas. The force was so great that the boy was knocked down. A purulent discharge was set up from which he never recovered. Dr. St. John Roosa in a letter to me states that he has never seen such a case, though he has heard of them.

A. H. Buck has found rupture of membrana tympani from concussion of air in external auditory canal very rare in his experience, only having recorded from his practice five cases. The cause of two being a box on the ear, two unknown and in the remaining one the patient being struck on the ear by a snow-ball. (A Manual of Diseases of the Ear, p. 345, Ed. 1889.) Arthur Hartmann mentions compression of air in the tympanum by sneezing, coughing and the employment of the air bag, but has nothing to say upon the subject in connection with syringing. (Die Krankheiten des Ohres, p. 120). Loud explosions, firing guns close to the ear and other concussions are prolific causes according to Wilhelm Kirchner and others. (Handbuch der Ohrenheil. Berlin, 1890, p. 78.) In the most of these cases it is very probable that there is some pathological change in the drum membrane itself, which has weakened its tissues and put it in condition to be more easily affected by these causes, such as calcareous deposits, old cicatrices, undue thinning or atrophy of the membrane as in chronic aural catarrh, etc.

The above case should teach us that we cannot be too careful in performing even this apparantly trivial operation of syringing out the ear. Very great force is not necessary, and I do not believe a very large and powerful syringe is needed. Some physicians have claimed that syringing could never do harm, provided only water was used, but I cannot agree with them. Frequently we see cases of ear disease only aggravated by the continuous use of this instrument. Only a short time since I saw a little sufferer with chronic catarrh of middle ear and eczema of canal, the latter produced by an almost daily syringing for a year. The physician had diagnosed wax accumulation and given the mother directions to syringe out ear until she removed all wax. This treatment she kept up faithfully, but she never saw the wax in the returning water, and finally, when the ears had become very painful the child was put under my care. After the usual treatment for aural catarrh, hearing was completely restored, but for a time the tendency to a recurrence of the eczema remained. While syringing is a good thing at times and under certain circumstances, yet I cannot council its indiscriminate use, and would advise that when it is necessary to use it, the greatest care and gentleness be exercised. In purulent troubles of the ear, usually one syringeful of water will be sufficient to remove all *débris*, and when there is impacted wax a weak solution of bicarbonate of soda poured in the ear will after several hours so soften it that it can be removed without nay difficulty.

